# Simultaneous Muraine Sutures and Excimer Laser-Assisted Penetrating Keratoplasty for Acute Keratoconus

**DOI:** 10.3390/jcm13133792

**Published:** 2024-06-28

**Authors:** Marie Elisabeth Burghardt, Joana Heinzelmann, Marlene Stein, Anja Viestenz, Arne Viestenz

**Affiliations:** Department of Ophthalmology, UMH, Martin-Luther-University Halle-Wittenberg, 06120 Halle (Saale), Germanymarlene.stein@uk-halle.de (M.S.); anja.viestenz@uk-halle.de (A.V.); arne.viestenz@uk-halle.de (A.V.)

**Keywords:** acute corneal hydrops, acute keratoconus, Descemet’s membrane, keratoconus, non-mechanical Excimer-Laser assisted penetrating keratoplasty, Muraine sutures, predescemetal sutures

## Abstract

**Background:** Acute keratoconus (acute KC), which affects approximately 1.6–2.8% of keratoconus (KC) patients, is a pathological condition of the cornea characterized by stromal edema due to entry of aqueous humor through a tear in Descemet’s membrane. **Methods**: We present a novel combination of surgical procedures that allows swifter visual recovery in a consecutive, retrospective case series. The new surgical procedure for acute KC consists of a combination of Muraine corneal sutures to smooth the corneal curvature and Excimer laser-assisted penetrating keratoplasty and was performed in six acute KC patients from 2019 to 2022 at the Department of Ophthalmology, University Hospital of Martin-Luther-University Halle-Wittenberg (UMH), Germany. We monitored data on preoperative status, operative details, intraoperative and postoperative complications and visual outcomes were analyzed. **Results**: The mean age was 41.5 **±** 13.5 years (3 OD, 3 OS). Neurodermatitis was present in 3 patients (50%). All patients received significant visual benefits from the procedure. Preoperative BCVA was hand motion (logMAR 3.0) in all patients; postoperatively, BCVA improved significantly logMAR 0.03 **±** 0.09 [range: 0.2–0.4; *p* < 0.001, FUP 20+/−10 months). Visual acuity remained stable throughout the roughly biannual follow-ups. One patient developed endothelial graft rejection after 2 years. During the last examination, all eyes had clear grafts and stable curvatures, K1 and K2 being 42.43 ± 4.17 D and 44.95 ± 4.07 D, respectively, and mean corneal astigmatism was 2.61 ± 1.74 D. The thinnest corneal thickness was 519 ± 31 µm. A graft size of 8.0 × 8.1 mm was the most beneficial. **Conclusions**: in patients with acute KC and hydrops, a penetrating keratoplasty with Muraine corneal sutures is successful in terms of graft clarity and visual outcome. Combining the procedures allows quicker visual recovery. Patients with a history of neurodermatitis should have preoperative and postoperative dermatologic treatment and close follow-up for possible complications.

## 1. Introduction

**Figure 1:**
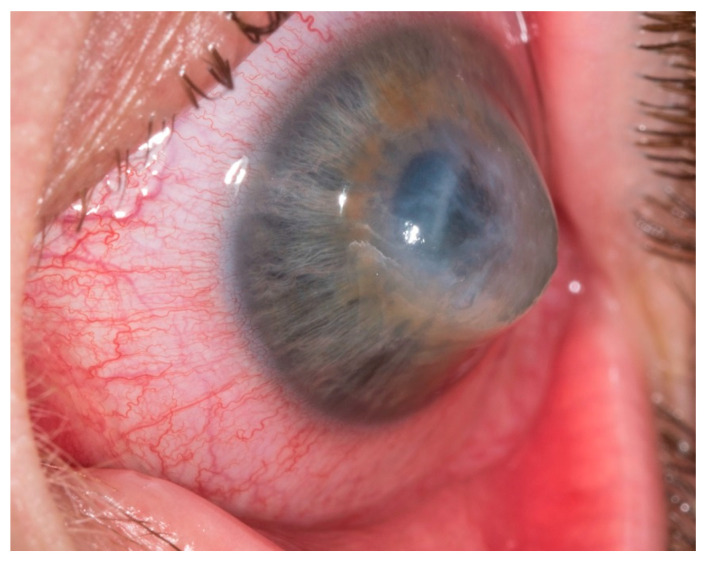
Corneal perforation in acute keratoconus with iris tamponade (Patient 3).

**Figure 2:**
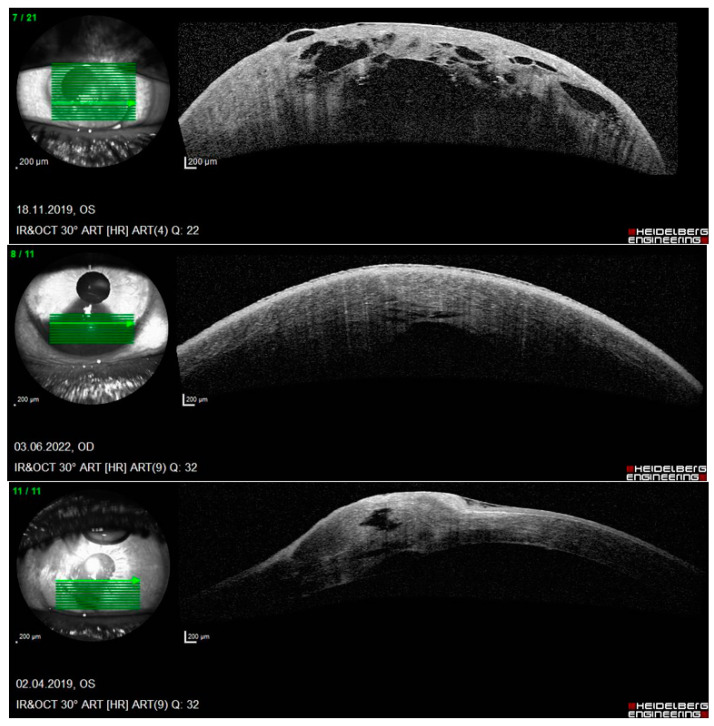
Anterior segment OCT: examples of three corneal perforations with tears in DM (Patient 3).

**Figure 3:**
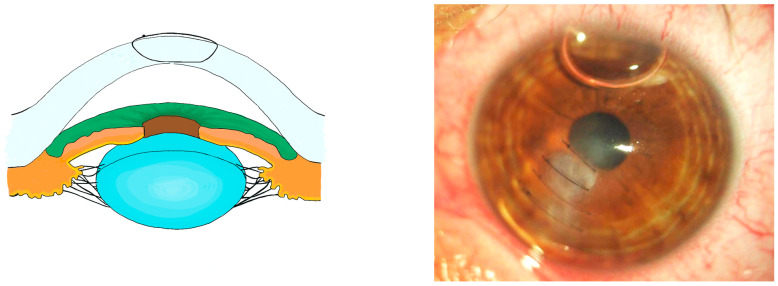
Schematic illustration of a predescemetal suture paired with a slit-lamp image of the same (Patient 2).

Keratoconus (KC) is a slowly progressive form of corneal ectasia, which usually begins to become symptomatic during puberty and stabilizes usually in the fourth decade of life. It is a bilateral and asymmetric disease that results in progressive thinning and steeping of the cornea, leading to irregular astigmatism, myopia, and decreased visual acuity. The prevalence and incidence rates of keratoconus have been estimated to be between 0.2 and 4790 per 100,000 persons and 1.5 and 25 cases per 100,000 persons/year [1]. It is characterized by an often progressive and irregular bulging and thinning of the cornea at the cone’s apex. This increased corneal steepening and distortion leads to myopia and irregular astigmatism and can result in superficial as well as deep stromal scarring [2]. In cases of excessive bulging, a tear in Descemet’s membrane (DM) can lead to the special form of “acute keratoconus (KC)” or “acute corneal hydrops”. This affects approximately 1.6–2.8% of patients with KC [1]. It describes a pathological condition of the cornea characterized by stromal edema due to the entry of aqueous humor through a tear in DM (Figure 1, Figure 2 and Figure 3). Young men around the age of 25 with advanced corneal ectasia are predominant [3]. Predisposing factors include excessive eye-rubbing, neurodermatitis, a history of conjunctivitis vernalis, allergy, and trisomy 21 [4]. A central corneal scar, restricting vision, often remains after the acute event, and surgical treatment may be required. In this special case, however, we are reaching our limits when using established standard techniques only. Penetrating keratoplasty is a promising treatment option since acute KC complicates lamellar surgery due to scars in the DM area, making the need for multiple procedures. It is currently agreed upon that a penetrating keratoplasty (PKP) should not be performed in the acute stadium of KC [5]. This is due to the altered corneal architecture caused by edema, which carries the risk of significant postoperative astigmatism. Due to the young age of the patients, collective rapid visual rehabilitation is a priority from a socioeconomic point of view. With this case series, we describe a novel combination of techniques—simultaneous corneal Muraine sutures [6] with penetrating excimer-laser-assisted keratoplasty—as an answer to the quest to find a faster and safer method of visual rehabilitation.

### Design

We present a consecutive, retrospective case series consisting of six patients. The small number of patients is due to the fortunate relative uncommonness of acute KC.

## 2. Materials and Methods

### 2.1. Setting

All procedures have been performed at the University Hospital of Martin-Luther-University Halle-Wittenberg in Halle (Saale), Germany, by the same experienced surgeon. Excimer-laser-assisted penetrating keratoplasty was performed with the Schwind Amaris^®^ 750S Model (Schwind eye-tech-solutions GmbH, Kleinostheim, Germany). Surgery was further performed under general anesthesia (intubation) with rocuronium as a muscle relaxant to minimize the phenomenon of Vis-à-tergo—a sudden swelling of the vitreous with a possible and much-feared complication of expulsive bleeding [7]. To further reduce Vis-à-tergo complications, controlled arterial hypotension was aimed for, and pupillo-constringent topical (pilocarpine eye drops 2%) was instilled every 15 min, 1 h before the procedure [8].

**Figure 4:**
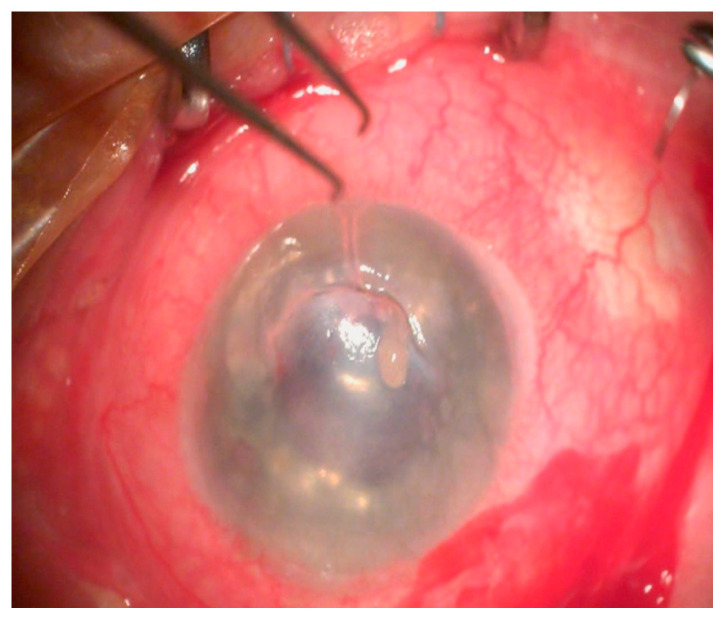
Intraoperative image of a ruptured cornea (Patient 3).

**Figure 5:**
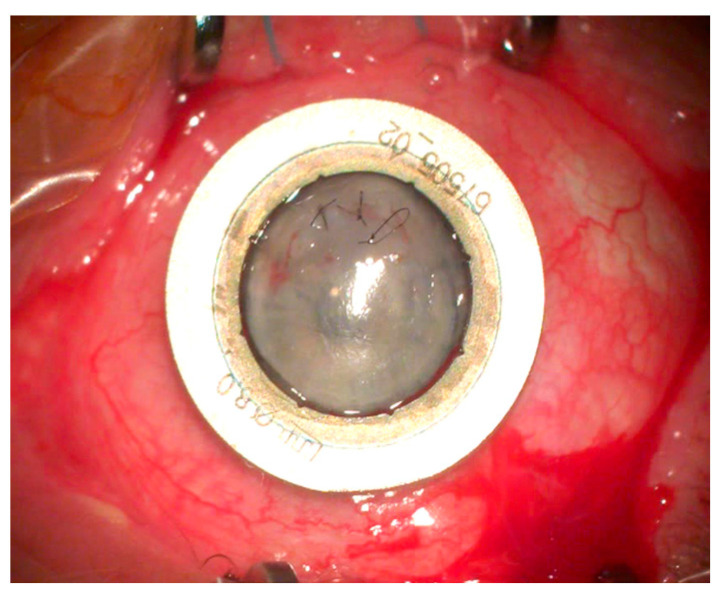
Excimer laser recipient mask after Muraine suture positioning (8.0 mm) (Patient 3).

**Figure 6:**
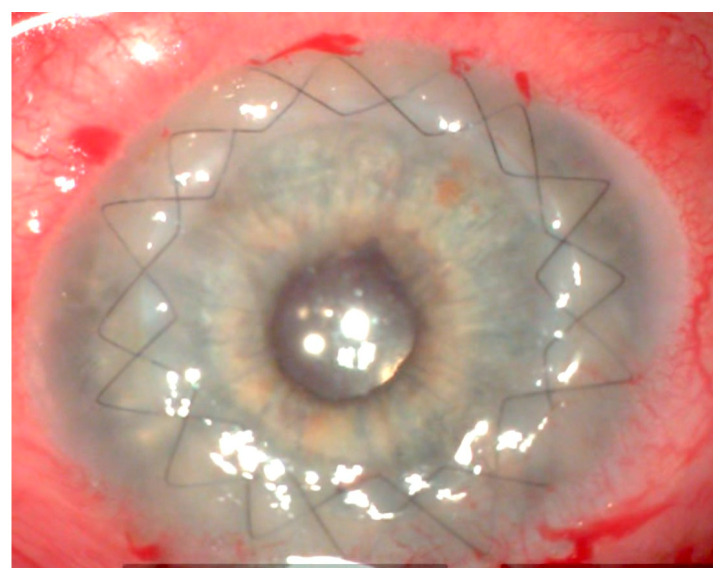
Immediate postoperative image (Patient 3).

To begin with, the inferior and superior rectus muscles were slung with a 3-0 polyethylene suture to eliminate the inadvertent Bell’s phenomenon. After intracameral air injection through a paracentesis, the cornea was left untouched for 10 min, and then deep stromal, predescemetal, singular 10-0 nylon sutures (Muraine sutures, Figure 4) were applied perpendicular to Descemet’s membrane tear (Figure 5). These predescemetal sutures facilitate the shaping of the cornea by suture tension and reduce the width of the DM tear mechanically. A Placido disc-light with 32 LED light sources was projected onto the corneal surface to evaluate the roundness of the corneal periphery (ZEISS OPMI Lumera 700, Germany). Once round, no further sutures were applied. Subsequently, air was again insufflated into the anterior chamber. Hyperosmolar eye drops for tissue dehydration were applied regularly throughout the entire process. The time used to prepare the graft in Excimer-laser-assisted trephination was used to continue the application of hyperosmolar eyedrops. The graft trephination was realized by placing the donor tissue in a modified artificial anterior chamber bench (after Krumeich) filled with viscoelastic gel (hyaluronic acid). Laser-assisted trephination was then performed along the outer borders of a round, open metal plate (diameter 7.6 mm or 8.1 mm; central opening 3.0 mm for centering; eight orientation teeth; 0.5 mm thickness; 0.2 g weight, Naumanns corneal trephination mask with 8 orientation teeth, Schwind, Kleinostheim, Germany) under microscopic surveillance. Pressure within the artificial chamber was aimed to be at approximately 22 mmHg. The remaining tissue bridges after trephination were separated with Katzin scissors. To prepare the laser-assisted trephination of the recipient’s cornea, viscoelastic gel (hyaluronic acid) was instilled into the anterior chamber, and a matching metal plate of 8.0 mm or 7.5 mm diameter and eight orientation notches (key–lock-principle) were centered on the cornea [8]. Upon the first corneal perforation, the laser ablation was stopped and the remaining stromal and Descemet’s tissue was separated again using Katzin scissors. A peripheral iridotomy was performed at the 12 o’clock position to avoid Urrets–Zavalia syndrome, a post-operative complication after PKP presenting with wide and rigid pupils, multiple posterior synechiae, and iris atrophy, and secondary glaucoma [9]. To secure the graft and stabilize the eye, eight cardinal sutures are applied, followed by a double-running suture (after Hoffmann [10]) to integrate the graft at best (Figure 6) with 10-0 nylon. A subconjunctival deposit of dexamethasone and gentamycin was applied, as well as immediate postoperative treatment with atropine 0.5%, prednisolone acetate eye drops, as and ofloxacin ointment. A closed wound dressing was applied. By default, intravenous injections of 500 mg Acetazolamide and 200 mg Prednisolone were administered.

The statistical analyses were performed with the program SPSS for Windows 22.0 (IBM SPSS Software, New York, NY, USA). The t-test, in conjunction with Levene’s test, was employed for these analyses. A *p*-value < 0.05 was considered to indicate statistical significance.

### 2.2. Study Population

We present six consecutive cases of acute KC treated with Muraine corneal sutures and a directly subsequent non-mechanical penetrating (Excimer-laser) keratoplasty from January 2019 to June 2022. All patients were male and had a job obligation (whether self-employed or employed). One of the patients was of critical value to his employer, being in charge of the computer-based administration of a power plant.

### 2.3. Observation Procedure

Data on preoperative status, operative details, intraoperative and postoperative complications, and visual outcomes, as well as Scheimpflug images (Pentacam, OCULUS Optikgeräte GmbH, Wetzlar, Germany) and OCT of the anterior segment (SD-OCT Spectralis, Heidelberg Engineering GmbH, Heidelberg, Germany), were archived for analysis. The BCVA pre- and postoperatively, as well as the final corneal curvature, were investigated.

## 3. Results

**Figure 7:**
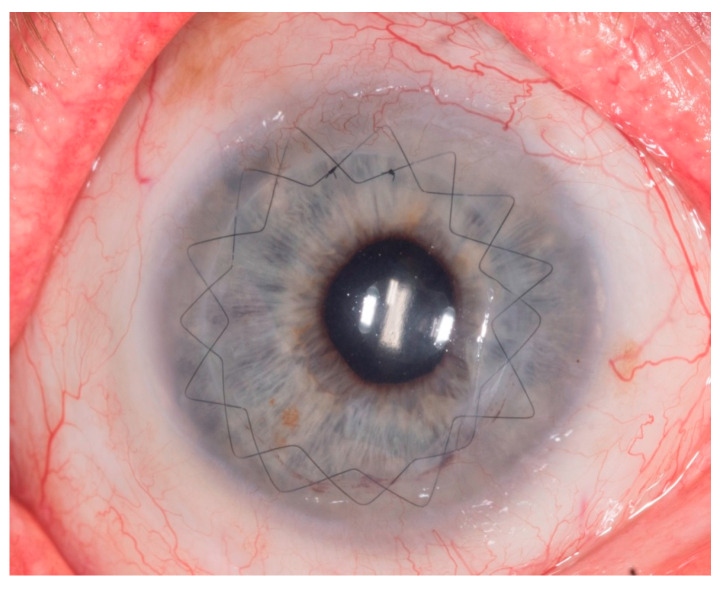
Postoperative slit lamp image 6 weeks after PKP (Patient 3).

The mean age in our group of patients was 41.5 ± 13.5 years. Three OD and three OS were affected. Neurodermatitis was present in 3 patients (50%); in no patient was Trisomy 21 was present. All patients were first seen in an emergency setting and admitted because of acute KC. They reported a pre-event visual acuity of the affected eye that corresponded more or less to that of the partner eye. The procedure resulted in significant visual benefits for all patients. Whereas the preoperative BCVA with underlying acute KC was hand motion (logMAR 3.0) in all patients, the best postoperative BCVA improved significantly (logMAR 0.30 ± 0.09 [range from 0.2 to 0.4, *p* < 0.001]). BCVA was entirely attained by spectacle correction, not rigid contact lenses. The longest follow-up period for one patient to date is 42 months (for reference, see Table 1). Visual acuity remained stable in all individuals throughout the follow-ups every 5–6 months except for one patient who developed an endothelial graft rejection after 2 years. His initial BCVA postoperatively was 0.6. After a Descemet membrane endothelial keratoplasty (DMEK) was performed, the BCVA could be stabilized at 0.3. Upon our latest examination, all eyes had clear grafts and stable curvatures. During the latest follow-up, Scheimpflug imaging showed a K1 of 42.43 ± 4.17 D and a K2 of 44.95 ± 4.07 D. The mean corneal astigmatism was 2.61 ± 1.74 D at last follow-up (range: 0.6 to 4.7 D) (see Table 2). The thinnest corneal thickness was recorded at 519 ± 31 µm. Due to the emergency status of acute KC, we unfortunately do not have preoperative keratometry data for all patients. Moreover, we were facing technical limitations of the Scheimpflug camera, which was unable to generate imaging due to the rough corneal surface in some patients. In three patients, we were successful in Scheimpflug imaging. These data showed preoperative curvatures of K1 at 61.5 ± 75.7 D and K2 at 67.5 ± 71.0 D. The lowest corneal thickness was 396 ± 248 µm (Table 3). Compared to the postoperative results, corneal stabilization and a reduction in astigmatism became apparent. A graft size of 8.0 × 8.1 mm has proven to be most beneficial (Figure 7).

## 4. Discussion

Acute KC is a relatively rare condition (1.6–2.8% of KC patients) [11]. Its uncommonness may be largely attributed to the intense research that has been conducted in KC by leading ophthalmologists and visionaries of our field during the last decades up to this date and the strict realm of regular control examinations that KC patients nowadays undergo. The latest introduction of the ABCD(E)-grading system is a sensible addition to help with decisions in stage-coherent therapy of KC patients [12,13]. In the early stages, therapeutic measures include prescription glasses and soft, or preferably rigid, oxygen-permeable contact lenses [14] for visual rehabilitation purposes. Scleral lenses form a potent alternative for intolerance of contact lens-wearing [15]. Another more invasive option is the implantation of intrastromal corneal ring segments (ICRS) [16]. An epithelium-off Riboflavin-UV-A-Crosslinking (CXL) is indicated in progressive KC cases with a central corneal thickness >400 µm and no central scarring [17]. The goal of CXL is to improve the stability of the cornea by cross-linking the stromal collagen fibers with riboflavin and under the influence of UV-A light [18]. Currently, the Dresden Protocol Guidelines are considered the gold standard, but promising therapeutic novelties include individually AI-software-tailored doses of Riboflavin and UV-A for each patient, as shown by Roszkowska et al. [19]. If successful, it might be possible in the future to perform CXL in patients with a central corneal thickness <400 µm by altering the treatment settings during the procedure based upon the individual corneal topography. For patients with higher stages of KC, featuring central corneal scarring and/or significant corneal thinning, treatment options include lamellar or penetrating keratoplasty (PKP). In patients with deep stromal scarring, including patients after acute KC, the therapy of choice is a PKP. Alternatively, a (laser-assisted) deep anterior lamellar keratoplasty (DALK) can be performed in patients with no pre-descemetal scars and a good functioning endothelium [20,21]. PKP in KC patients is an intricate microsurgical endeavor that requires planning ahead of surgery and continuous reevaluation of the situation during the treatment. An important issue to consider is the postoperative astigmatism. Even in less challenging topographical corneas astigmatism control can be a hardship for the (in)experienced surgeon. Intraoperatively the three most significant determining factors for the postoperative outcome in astigmatism, according to Seitz et al. (2013) are: the decentralization of the recipient’s trephination area, the horizontal torsion and vertical tilting of the graft [22]. Therefore, the patient’s head and limbal region should be positioned perfectly aligned with the horizon to ensure an optimal centering of the trephination mask. Due to the irregularity of the cornea in KC, the surgeon cannot take the optically misplaced pupil as a reference point for the centering of the trephination mask but instead must use the limbus [23]. Once centered, another important issue is the compression and distortion or tilt of the corneal tissue during mechanical trephination. In 1989, Excimer-laser-assisted PKP, first described by G.O.H. Naumann and G.K. Lang et al., revolutionized PKPs in KC as a method of noncontact trephination [24]. The eight orientation teeth and notches that were included in the metal trephination masks and form a powerful guiding structure to place sutures correctly and with the least astigmatic power [25]. In combination with a Hoffmann double-running corneal suture [10], a significant reduction in postoperative corneal astigmatism could be achieved [26]. Excimer-laser-assisted PKP has proven to be superior to femtosecond-laser-assisted PKP in both postoperative astigmatism and visual rehabilitation [27].

Acute KC poses challenges for the surgeon because of the partially enormous edematous swelling of the corneal tissue due to the influx of anterior chamber fluid into the corneal stroma. It is a current agreement that a penetrating keratoplasty during an acute KC should not be performed. Complications such as the risk of suture loosening with subsequent infiltrates, stromal vascularisations, and even transit endophthalmitis are cited [22]. Up until a few years back, the treatment of acute KC consisted mainly of applying hypersaline eye drops (natriumchloride 5%) as well as “sit and wait” until after 3–6 months, a spontaneous healing (scarring) is achieved [22].

Currently, a technique of predescemetal sutures and intracameral gas injection, which was first described by H.Y. Chérif et al. from the research group of Prof. Marc Muraine of Rouen University, France, in 2015, has gained favor [6]. The so-called “Muraine sutures” aim to mechanically readapt the ruptured margins of Descemet’s tear by placing sutures perpendicular to the tear. It is crucial for their function to go deep into the stroma, at best pre-descemetal, which means at least 90% of the corneal thickness. These sutures, in combination with intracameral gas injection, have proven beneficial for stabilizing acute corneal hydrops and significantly reducing corneal edema [28]. The instillation of gas into the anterior chamber shall prevent the influx of further fluid into the corneal stroma. Instillation of expanding Perfluorpropane (14% C3F8) seems to be more efficient (8.8 ± 4.9 days) than Sulfur Hexafluoride (SF6) (4 weeks) and air injection (20.1 ± 9.0 days) [28,29,30]. Over time, a penetrating keratoplasty may follow [5]. The disadvantage of this method is the visual impairment for a period of at least 6 months until a PKP is performed. Patients may thus not be able to perform their work, drive a car, or engage in sports for a longer period of time. The Keratoconus Outcomes Research Questionnaire (KORQ) has shown that quality of life declines with worse visual acuity [31].

We found, that in addition to these anti-edematous effects, Muraine sutures also hold a powerful impact on shaping the cornea. Provided the tear in DM is paracentrally, leaving the corneal margins largely unaffected, Muraine sutures can be used to reduce the astigmatic effect of the ruptured cone by compression. Combined with the technique of gas instillation into the anterior chamber as well as the application of hypersaline eye drops throughout the first part of surgery up until trephination, we have found that a PKP in acute KC is manageable. The combined surgical technique we describe allows immediate non-mechanical (Excimer-laser-assisted) PKP within the same session, avoiding a long interval between Muraine sutures and corneal transplantation. Visual rehabilitation may be accelerated by weeks (with Muraine sutures) or months (without intervention). However, it is of crucial importance for the tear to spare the periphery of the cornea. Fortunately, due to the usual cone position, the locus minoris resistenciae is commonly inferno-centrally.

For this surgical approach, we consider non-mechanical Excimer-laser-assisted PKP superior to mechanical trephination used in conventional keratoplasty or Femtosecond laser-assisted PKP. Advantages lie in the more precise method of trephination by non-mechanical excimer-laser-assisted photoablation and the ability to abstain from physical manipulation of the fragile tissue, resulting in the anti-astigmatic advantages discussed earlier [26,32]. Potentially, the softer, swollen tissue in the margin areas of the edema might compromise Excimer-laser-assisted PKP. A sufficient laser trephination in these areas might be harder to acquire.

In consequence, we are able to report an acceptable resulting postoperative astigmatism (2.6 D). This is less than astigmatism after corneal trephination with Excimer laser in non-acute stages of KC with Excimer laser (3.3 D) or Femtosecond laser (6.8 D) [32]. We can report a good visual outcome of logMAR 0.3. So far, we have not experienced any adverse event that would have compromised the decision to perform PKP.

A penetrating keratoplasty after an acute KC seems to be advantageous also in terms of corneal endothelial changes. These changes include cell pleomorphism, polymegathism, cell degeneration, and inflammation that might induce endothelial failure over time [33]. In one patient, a DMEK had to be performed two years after the initial PKP with Muraine sutures due to endothelial failure. We could not determine the cause for endothelial failure in this case, but after performing DMEK we found the cornea to remain clear.

Patients with a history of neurodermatitis/atopic dermatitis should have preoperative and postoperative dermatologic treatment and close follow-up for possible complications. They should be encouraged to refrain from rubbing the eye by any means. In patients with Trisomy 21, the indication for penetrating surgery should only be made after careful consideration and in close consultation with the caregiver. Although we have not had a patient with Trisomy 21 that would have been eligible for our combined surgery approach, we believe that our method would also be advantageous, provided the above criteria (placement of Descemet’s rupture) are met.

It should further be emphasized that all of our reported surgical outcomes can be attributed to one experienced surgeon. In the hands of lesser experienced clinicians more complications might occur.

There are limitations to our reported data. Due to the rareness of acute KC, we can unfortunately only review a small number of treated patients and hope to be able to report more cases and a longer history of follow-ups in the future. We would like to further investigate the intraoperative effects of Perfluorpropane (C3F8 gas) vs. Sulfur Hexafluoride (SF6 gas) and hope that with a growing number of treatments, we will be able to maintain a low resulting astigmatism. Our relatively young patient population makes the possibility of graft failure due to immunological and non-immunological reasons another important pillar in therapeutic decision-making. Our current follow-up time of a maximum of 42 months does not yet allow a definitive statement about the long-term outcomes of our procedure and the impact on future possible necessities of replacement grafts. A comparison between those long-term outcomes versus more conservative more conservative approaches has yet to be evaluated.

In summary, pre-shaping the cornea with Muraine sutures in patients with acute KC, followed by a non-mechanical Excimer-laser-assisted PKP, allows us to describe a promising combined technique that may improve the overall outcome of PKP quickly. It results in a better visual outcome with good results in graft clarity, faster visual rehabilitation, and a marked reduction in complications, leading overall to a higher quality of life, as suggested in the Keratoconus Outcomes Research Questionnaire (KORQ) [31]. For our future endeavors in this field of research, we plan to include a similar questionnaire in our assessment.

## Figures and Tables

**Table 1:** Patient data.

Patient	VA Pre-OP	VA Post-OP	VA Partner Eye	Age	Lateralization	Neurodermatitis	Diameter PKP [mm]	Date of Surgery	Last FUP
1	HM	0.4	0.6	57	OD	Unknown	8.0 × 8.1	8/2020	7/2022
2	HM	0.6	0.2	29	OS	Yes	8.0 × 8.1	4/2019	10/2022
3	HM	0.6	0.3	58	OD	Yes	8.0 × 8.1	1/2019	9/2022
4	HM	0.5	0.5	28	OS	Unknown	8.0 × 8.1	8/2020	4/2022
5	HM	0.5	0.6	43	OD	Yes	8.0 × 8.1	6/2022	9/2022
6	HM	0.4	1.0	34	OS	Unknown	7.5 × 7.6	10/2020	10/2021

Abbreviations: OD = Oculus Dexter (right eye); OS = Oculus Sinister (left eye); HM = Hand Motion; VA = Visual Acuity; PKP = Penetrating Keratoplasty; FUP = Follow-Up; OP = Operative.

**Table 2:** Postoperative keratometry data from Scheimpflug imaging at the latest follow-up.

Patient	K1 [D]	K2 [D]	Follow-Up [months]	Thinnest Corneal Thickness [µm]
1	43.5	44.0	16	534
2	42.6	47.3	42	574
3	47.6	49.3	33	488
4	42.7	43.5	23	503
5	34.8	38.0	12	499
6	43.4	47.6	20	516

Abbreviations: K = Keratometry; D = Diopters.

**Table 3:** Preoperative keratometry data from Scheimpflug imaging.

Patient	K1 [D]	K2 [D]	Thinnest Corneal Thickness [µm]
1	92.4	96.4	461
2	45.9	50.4	431
3	46.9	55.8	296

Abbreviations: K = Keratometry; D = Diopters.

## Data Availability

No new data were created.
